# The Interaction between Amyloid Prefibrillar Oligomers of Salmon Calcitonin and a Lipid-Raft Model: Molecular Mechanisms Leading to Membrane Damage, Ca^2+^-Influx and Neurotoxicity

**DOI:** 10.3390/biom10010058

**Published:** 2019-12-29

**Authors:** Marco Diociaiuti, Cecilia Bombelli, Laura Zanetti-Polzi, Marcello Belfiore, Raoul Fioravanti, Gianfranco Macchia, Cristiano Giordani

**Affiliations:** 1 Centro Nazionale Malattie Rare, Istituto Superiore di Sanità, I-00161 Roma, Italy; marcello.belfiore@iss.it (M.B.); raoul.fioravanti@uniroma1.it (R.F.); 2 CNR-Istituto per i Sistemi Biologici, UOS di Roma, c/o Dipartimento di Chimica, Sapienza Università di Roma, I-00185 Roma, Italy; cecilia.bombelli@cnr.it; 3 Dipartimento di Fisica e Scienze Chimiche, Università dell’Aquila, via Vetoio (Coppito 1), 67010 L’Aquila, Italy; laura.zanettipolzi@univaq.it; 4 Dipartimento di Chimica, Sapienza Università di Roma, I-00185 Roma, Italy; 5 Centro Grandi Strumentazioni e Core Facilities, Istituto Superiore di Sanità, I-00161 Roma, Italy; gianfranco.macchia@iss.it; 6 Grupo Productos Naturales Marinos, Facultad de Ciencias Farmacéuticas y Alimentarias, Instituto de Física, Universidad de Antioquia, Calle 70 No. 52-21, Medellín 050010, Colombia; cristiano.giordani@udea.edu.co

**Keywords:** amyloid proteins, neurotoxicity, lipid-rafts, GM1, cholesterol, salmon calcitonin, circular dichroism, transmission electron microscopy, ca^2+^-influx

## Abstract

To investigate the interaction between amyloid assemblies and “lipid-rafts”, we performed functional and structural experiments on salmon calcitonin (sCT) solutions rich in prefibrillar oligomers, proto- and mature-fibers interacting with liposomes made of monosialoganglioside-GM1 (4%), DPPC (48%) and cholesterol (48%). To focus on the role played by electrostatic forces and considering that sCT is positive and GM1 is negative at physiologic pH, we compared results with those relative to GM1-free liposomes while, to assess membrane fluidity effects, with those relative to cholesterol-free liposomes. We investigated functional effects by evaluating Ca^2+^-influx in liposomes and viability of HT22-DIFF neurons. Only neurotoxic solutions rich in unstructured prefibrillar oligomers were able to induce Ca^2+^-influx in the “lipid-rafts” model, suggesting that the two phenomena were correlated. Thus, we investigated protein conformation and membrane modifications occurring during the interaction: circular dichroism showed that “lipid-rafts” fostered the formation of β-structures and energy filtered-transmission electron microscopy that prefibrillar oligomers formed pores, similar to Aβ did. We speculate that electrostatic forces between the positive prefibrillar oligomers and the negative GM1 drive the initial binding while the hydrophobic profile and flexibility of prefibrillar oligomers, together with the membrane fluidity, are responsible for the subsequent pore formation leading to Ca^2+^-influx and neurotoxicity.

## 1. Introduction

It has been proposed that the protein misfolding process can involve practically all protein and considered as their “dark side” [1]. A number of highly diffused (Alzheimer’s, Parkinson’s and Type II Diabetes) or rare (Creutzfeldt–Jacob’s or Niemann–Pick’s) human diseases are linked to the misfolding of the involved proteins, the most famous amyloid-β (Aβ), α-synuclein (αS), amylin (hIAPP) or prion (Pr) [2,3].

There is a common self-assembly phenomenon, called amyloid aggregation, starting from the soluble misfolded proteins and leading to the formation of the typical amyloid fibrils [4,5].

**Figure 1:**
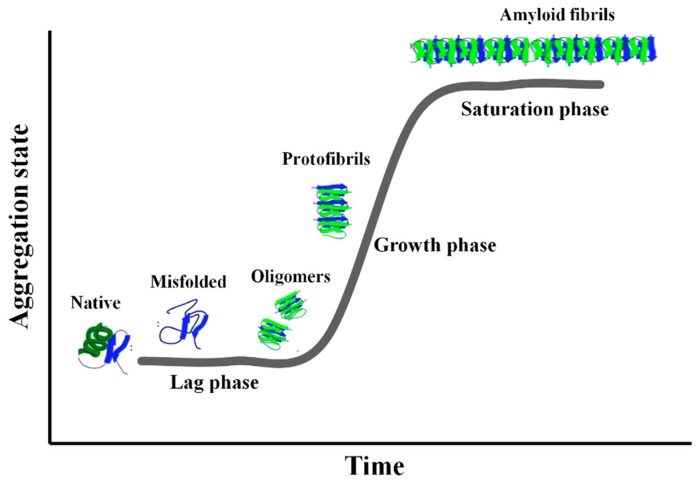
The typical aggregation curve of the amyloid proteins is characterized by three zones: “Lag-phase”, “Growth-phase” and “Saturation-phase”. The structure of the aggregates is also depicted (for the courtesy of Iannuzzi et al. [6]).

This behavior is characterized by a typical sigmoidal curve (Figure 1) showing an initial “Lag-phase”, where monomers form oligomers of low molecular-weight (i.e., dimers, trimers, tetramers, etc.) named prefibrillar oligomers (PFOs), generally characterized by the random coil (RC) configuration. A “Growth-phase” follows, where the formation of annular or linear protofibrils (APFs or LPFs) begins with the appearance of few β-structures. Finally, a “Saturation-phase” occurs, where insoluble mature fibers (MFs) characterized by the β-sheet configuration, are dominant. Recently, Arosio et al. have shown that the Lag- and “Growth-phase” do not correspond in a simple manner to the molecular events of nucleus (PFOs) formation and fibril (PFs) elongation, but that each phase results from a combination of events involving primary nucleation, fibril elongation, secondary nucleation and fibril fragmentation [2,4]. Thus, each phase contains all aggregates with relative concentration depending on the rate constants governing the interchanges [4]. The “Lag-phase” results to be mainly populated by PFOs and few PFs while the “Growth-phase” by PFOs, generated through secondary nucleation and fragmentation of the PFs [4], and an increasing percentage of PFs. The extension of the “Lag-phase” strongly depends on the protein primary sequence, concentration, temperature, ionic strength and pH [4].

It is now generally agreed that, among all aggregates of proteins involved in pathologies, the toxic species are the soluble PFOs while the insoluble fibrillar conformations (APF, LPFs and MFs) seem to be relatively non-harmful [2,3,7,8,9,10]. A particular Aβ oligomer of 56 kD, corresponding to a dodecamer and named Aβ*56, has been indicated as the direct responsible for the Alzheimer’s disease (AD) in *in vivo* models [11]. Kayed et al. showed that PFOs of Aβ_1–40_, Aβ_1–42_ and α-Synuclein were toxic while APFs were non-toxic distinct type of oligomers [12]. More in particular, Ono et al. demonstrated that, among Aβ-PFOs, trimers and tetramers were the toxic species [13]. For what concerns the causes, Campioni et al. demonstrated that the toxicity of two types of HypF-N oligomers was correlated to their low degree of hydrophobic packing and that structural flexibility and hydrophobic exposure were primary determinants of their ability to cause cellular dysfunctions [14].

However, as recently pointed out by Benilova et al., the tendency of proteins such as Aβ to rapidly aggregate during the experiments leads to a difficult and uncertain identification of the structure responsible for a definite biologic effect [15]. Thus, stable amyloid proteins not related to diseases or even synthetic were used to study the oligomer formation and interaction with lipid membranes [3,16,17]. The use of these proteins is also important to test the hypothesis of the existence of a common mechanism of action to all amyloid proteins, independent from the primary sequence [1,3,8,16,18].

Interestingly, since 2003 Glabe et al. proposed that a common core of pathologic pathways exists for amyloid-associated diseases, based on the cellular membrane permeabilization and subsequent abnormal Ca^2+^-influx, independent from the primary sequence of the involved protein [8,18,19,20]. Lashuel and Lansbury summarized the existing supportive circumstantial evidence about the hypothesis that amyloid diseases were caused by protein aggregates that mimic bacterial pore-forming toxins, which in general form well-ordered oligomeric membrane-spanning pores characterized by β-sheet structure [21]. There are many papers in literature concerning the formation of permanent pores in model membranes by several proteins, even not involved in pathologies. The mechanism of action has been reviewed for α-Hemolysin and occurred in a stepwise manner, from the soluble monomeric structure to the final membrane-embedded heptameric β-barrel pore [22]. Notably, a common molecular mechanism of pore formation by Aβ and α-sin involving gangliosides and cholesterol (Chol) has been proposed by Di Scala et al. [23]. More recently, Lee et al. reported on ion channels formed by Aβ in membranes comprising brain total lipid extract [24].

Emerging evidences are focusing on the neuronal membrane composition and its chemical micro-environment. It has been shown that the brains of AD patients contained increased fractions of anionic lipids, such as phosphatidylserine and phosphatidylglycerol [25,26] and that anionic lipids and not natural lipids favored cationic influx induced by Aβ [27]. It is now generally accepted that “lipid-rafts”, which are ordered nanodomains formed by sphingolipids (sphingomyelins and glycosphingolipids) and Chol abundant in the outer leaflet of the plasma membrane, play a special role [28,29,30]. Despite the extremely small size, within “rafts” are concentrated receptors and membrane proteins and in these domains are formed lipid–protein complexes whose formation would be impossible in the fluid phase consisting the “sea” of phospholipids. To “rafts” were attributed important functions such as signal transduction, membrane trafficking, neuronal differentiation and the entry of pathogens and toxins into the cell [29] and they have been associated with several neurodegenerative diseases. Notably, it has been reported their association with Aβ and assumed that this binding acts as a catalyst for the formation of neurotoxic aggregates [31,32].

Among glycosphingolipids, gangliosides are important for the role played in a number of important cellular functions, and in particular in neurons [33]. Monosialosilganglioside-GM1 (GM1), known as the most popular “raft-marker” [28,32,34], contains four neutral sugar moieties and a negatively charged sialic acid residue, displaying a net negative charge at physiologic pH [35]. It has been indicated to play a key-role in the interaction between amyloid proteins and cellular membranes [25,36,37,38,39]. Ikeda et al. proposed that GM1 clusters occurring in the “lipid-rafts” mediated the formation of the toxic fibrils at the membrane surface [40]. Matsubara et al. showed that GM1 density was a crucial factor for the assembly of Aβ-protein on detergent-resistant membrane microdomain fraction of synaptic plasma membranes of aged mouse brain, found that high-density GM1 nanoclusters were formed in the membrane and were closely connected with the formation of the spherical Aβ aggregates. Therefore, they concluded that a GM1 critical density was able to induce ganglioside-Aβ binding, acting as an endogenous seed for Aβ assembly in AD’s brains [32].

Last but not least, as recently pointed out by Sciacca et al. for Aβ and hIAPP, it is important to consider that the amyloid-membrane interaction can take place in a very short time (seconds) and that the time required to prepare samples and perform experiments (hours) did not allow studying the early stages [25]. For this reason, molecular dynamics (MD) simulations concerning the amyloid aggregate structures and interaction with membranes have been developed [41,42,43,44]. Notably, Shafrir et al. published MD simulations showing that Aβ hexamers formed transmembrane pores characterized by hexagonal symmetry [44].

Based on the previous considerations, in the past we proposed to use salmon calcitonin (sCT) as amyloid model [5,33,45]. sCT is particularly favorable due to the high solubility in water and very low aggregation rate that allows preparing, avoiding cross-linking procedures, stable solutions rich in selected oligomers. Working with cell lines we found that the neurotoxicity of aggregated and unfractionated sCT samples was similar if not greater than that of Aβ and correlated to the abundance of “lipid-rafts” in the cells [46]. Thus, to investigate this outcome we performed biophysical studies on the interaction of sCT with model membranes mimicking the presence of “lipid-rafts” confirming the GM1 pivotal role [47]. Recently, we fractionated the sCT aggregates and demonstrated that PFOs was the species able to induce neurotoxicity in primary hippocampal neurons and mouse brain slices [5,20].

However, up to now the nature of the forces driving the interaction of sCT-PFOs with “lipid-rafts” and the membrane properties determining the damage, are not clear. To investigate this matter we will use sCT as amyloid model that shows, at pH 7.4, an overall positive charge 3+ (isoelectric point is 10.4) and liposomes mimicking the sole “lipid-rafts” and not the whole cellular membrane [48]. For comparison we will use liposomes with and without GM1, which affects the membrane charge, and with and without Chol, known to affect membrane fluidity.

By exploiting the very slow aggregation rate of sCT, we will prepare native and stable solutions incubating it to obtain solutions rich in: PFOs when the incubation ends in the “Lag-phase”, mixed populations of PFOs and PFs in the “Growth-phase” and rich in MFs in the “Saturation-phase”. All solutions will be characterized by the combined application of size exclusion chromatography (SEC), energy filtered-transmission electron microscopy (EF-TEM) and circular dichroism (CD).

To focus on the role played by the electrostatic charges present at the membrane surface, we will compare results relative to GM1-containing liposomes mimicking “lipid-rafts”, made of DPPC/Chol/GM1 (48:48:4 mol%), with those relative to the zwitterionic DPPC/Chol (50:50 mol%) and plain DPPC systems. To focus on the effects due to the membrane fluidity, the comparison will be made with Chol-free liposomes made of DPPC/GM1 (96:4 mol%) and plain DPPC. In our case, DPPC/Chol/GM1 represents the “raft” model and GM1 plays the role of sphingomyelin. In fact, both belong to the sphingolipid family and share the ceramide backbone, fundamental to simulate the sole *l_o_* phase of “lipid-rafts, even though GM1 is negatively charged. The permeabilization (i.e., Ca^2+^-influx) induced at room temperature (RT) by the sCT solutions, will be evaluated by fluorescence experiments.

To correlate permeabilization and neurotoxicity, viability experiments will be performed on HT22-DIFF neurons treated with the sCT solutions. Finally, to study the sCT configuration and liposome morphology evolution during the interaction, structural studies will be performed by CD and EF-TEM techniques.

## 2. Materials and Methods

### 2.1. Materials

1,2-Dipalmitoyl-*sn*-glycero-3-phosphocholine (DPPC; MW 735 Da; purity > 99%) was purchased from Avanti Polar Lipids (Alabaster, AL, USA) monosialoganglioside-GM1 (MW 1545 Da) from bovine brain, cholesterol (MW 387 Da), Magainin II (MW 2467 Da), HEPES, EPPS, EGTA, CaCl_2_ (99%), KH_2_PO_4_, Na_2_HPO_4_ and all organic solvents were purchased from SIGMA-Aldrich (SIGMA Chemical, St Louis, MO, USA). Lyophilized sCT (MW 3432 Da) was purchased from European PHARMACOPOEIA (EDQM, Strasbourg, FR) and stored at −18 °C before use. FLUO-4 was purchased from Molecular Probes, Inc. (Eugene, OR, USA). All the chemicals were used without further purification. Phosphate buffer (PB; 5 mM, pH 7.4), or HEPES/EGTA buffer (5 mM/0.1 mM pH 7.4), were used. Milli-Q water was produced by a Direct-Q3 Millipore apparatus (MERCK KGaA, Burlington, MA, USA) and was used for all aqueous solution preparation.

### 2.2. Sample Preparation

To eliminate preformed sCT aggregate, 1.1 mg of sCT was dissolved in 320 µL of hexafluoroisopropanol (HFIP) to reach the final concentration of 1 mM [46]. HFIP was eliminated by evaporation using a Rotavapor at 35 °C and pression of 8 mmHg. The film formed was subjected to a pressure of 10^−3^ mmHg for 3 h to eliminate HFIP residues. Dried film was stored at −20 °C before further use. This film was subsequently rehydrated in PB. Initially, to the round-bottom flask containing the film were added 2 mL of PB. The solution so obtained was placed in an ultrasonic bath for about 10 min to dissolve completely the protein film. The sonicated solution was further diluted in PB until a final volume of 25 mL. The concentration of protein in PB is then 13 μM, according to the values in literature for obtaining a population of oligomers [49]. The solution thus obtained is stored at 4 °C, in order to reduce the aggregation rate [49], for increasing times (t = 0, 1, 9, 15 and 21 days). Magainin II was directly dissolved in PB at a final concentration of 13 μM.

For liposome preparation, four different lipid films were prepared: (1) DPPC/Chol/GM1 (48:48:4 mol%); (2) DPPC/Chol (50:50 mol%); (3) DPPC/GM1 (96:4 mol%) and (4) DPPC by mixing the proper amounts of DPPC, Chol and GM1 solutions in CHCl_3_/CH_3_OH/H_2_O 2:1:0.15. The obtained solutions placed in 100-mL round-bottomed flasks were connected with a rotary evaporation unit and the solvent was evaporated to form lipid films that were dried by a vacuum pump overnight. After drying, the films were rehydrated at 325 K with the proper buffer (PB for CD and EF-TEM analyses and HEPES/EGTA for fluorescence experiments, in the presence or in the absence of FLUO-4 50.3 μM) in order to obtain a 2.5 mM (total lipids) dispersion. The dispersions were vortex mixed and then frozen thawed six times from liquid nitrogen to 335 K; finally they were extruded (10 times) through a 100-nm polycarbonate membrane (WHATMAN NUCLEOPORE, Clifton, NJ, USA). The extrusions were carried out at 335 K, well above the transition temperature of DPPC (315 K), using a 2.5-mL extruder (LIPEX BIOMEMBRANES, Vancouver, Canada). Liposome size was evaluated diluting the sample with buffer (0.5 mM total lipid concentration) by dynamic laser light scattering (DLS) using a NanoZetasizer apparatus (MALVERN PANALYTICAL, Malvern, UK), equipped with a 4 mW HeNe laser source (k = 632.8 nm). In this apparatus, the light scattered by the sample placed in a thermostatted cell-holder, was collected at an angle of 173°. Liposome dispersions (2.5 mM) were diluted 1:10 with buffer for CD and EF-TEM analyses.

### 2.3. Size Exclusion Chromatography (SEC) Characterization

Native sCT solutions were loaded in the SEC column in order to characterize its content. Briefly samples were loaded in SEPHADEX G100-SEC column (GE HEALTHCARE, Milano, Italy. Height: 370 mm, section: 10 mm). Column, maintained at 4 °C was pre-equilibrated at the same ionic strength of the samples and calibrated with a solution containing standards: aprotinin 1 mg (MW 6511 Da), cytochrome C 1 mg (MW 12,400 Da-COMBITHEK BOEHRINGER, Mannheim, Germany) and somatostatin 1 mg (MW 1638 Da) suspended in 5 mM PB buffer pH 7.4, and centrifuged at 15,700× *g* 10 min. Aggregated sCT solutions (0.5 mL aliquots), prepared as descripted above, were eluted in the column monitoring absorption at 280 nm by a variable wavelength UV detector (BIORAD Econo UV monitor, Hercules, CA, USA). In order to exchange the buffer for the permeabilization experiments, sCT aggregates (13 μM) incubated in PB at 4 °C for increasing times (t = 0, 1, 9, 15 and 21 days) were filtered on SEC columns. PB was removed on SEPHADEX G-25 (medium) minicolumns preequilibrated with HEPES/EGTA using the dry filtration protocol [50]. Briefly, 400 μL of sCT aggregate solutions in PB were charged on 2.5 mL minicolumns and centrifuged at 1000× *g* 3 min; then 450 μL of HEPES/EGTA buffer were loaded on each column and centrifuged in the same conditions. Protein concentration in eluate solution was evaluated by the Bradford method. Free FLUO-4 non-entrapped in liposome aqueous core, was removed by filtration on SEPHADEX G-50 minicolumns preequilibrated with HEPES/EGTA (GE HEALTHCARE, Milano, Italy), using the dry filtration protocol [50]. Briefly, 200 μL of liposome dispersions were charged on 5 mL minicolumns and centrifuged at 1000× *g* 3 min. The presence of liposomes in the eluate was verified by DLS measurements.

### 2.4. Liposome Permeabilization

Ca^2+^-influx was evaluated by fluorescence emission experiments exploiting FLUO-4, embedded in the liposome core, as Ca^2+^ indicator dye. Steady state fluorescence spectra were recorded at RT on a HORIBA JOBIN-YVON Fluoromax 4 spectrofluorometer (Kyoto, Japan). Spectra were corrected by means of a built-in program in order to counterbalance the decay in sensitivity in the near infrared region. All fluorescence experiments were carried out on solutions with optical density lower than 0.05 to minimize the inner filter effect.

Samples were prepared by adding 800 μL of each sCT solution to 200 μL of FLUO-4 containing liposome dispersions (DPPC/CHOL/GM1, DPPC/CHOL, DPPC/GM1 and DPPC) and by allowing the aggregates to interact with the liposome membrane for 24 h. As positive control was used Magainin II (Mag. II), a well-known pore forming antimicrobial peptide, that was allowed to interact with the three liposome formulations, at 13 μM final concentration for 24 h. Immediately before the fluorescence measurement, each solution was diluted with HEPES/EGTA for aggregated samples (final volume 2 mL) in a quartz cuvette with a 1 cm path-length.

Fluorescence measurements were performed by monitoring the intensity change in FLUO-4 fluorescence emission (λ_exc_ = 494 nm, λ_max_ = 516 nm) after the addition of 20 μL of a CaCl_2_ solution (17.2 mM in buffer) to the cuvette, to obtain a 172 μM final Ca^2+^ concentration. The fluorescence increment upon Ca^2+^-influx in each sample was determined as it follows:

(1) $$F_{\mathrm{inc}}=\frac{F-F_{0}}{F_{0}}\times 100$$

where F_0_ is the fluorescence of liposomes and sCT before the addition of Ca^2+^ and F is the fluorescence after the addition of Ca^2+^.

### 2.5. Circular Dichroism (CD) Spectroscopy

Samples were analyzed by CD measurements performed on a JASCO J-715 spectropolarimeter (Tokyo, Japan) in the far-UV region (260–190 nm). All spectra, mean of four different scans, were blank subtracted. Quartz cells of 0.1 and 0.5 cm pathlength were employed. The spectral resolution was 0.5 nm and the speed scan 50 nm/min. All CD spectra were reported as:

Δε = [Θ]/3300,
(2)

where:

[Θ] = (θ × 100)/(l × c),
(3)

(θ is the measured ellipticity, c is the molar amino acidic concentration and l is the pathlength in cm).

CD spectra obtained from solutions where protein aggregates of size comparable to the light wavelength are present, are affected by two phenomena: the “scattering” and the “flattening”. The first, due to the incoherent diffusion of the incident light, decreases the intensity of the detected signal and create an apparent rise of the absorption. Conversely, the second, due to the concentration of the chromophores in the aggregates, decreases the probability that the photons were scattered and introduces the lowering of the absorption [51].

Under our experimental conditions, the “scattering” phenomenon can not be eliminated because the impossibility to modify the acquisition geometry. However, it can be quantitatively estimated at all wavelength by acquiring, from the same solution, an absorption spectrum at λ > 310 nm. We checked that the “scattering” was negligible before proceeding with the CD analysis. When this condition was not verified the obtained results represents only a qualitative estimation of the protein conformation, useful to integrate the general trends of the study.

In the absence of “scattering”, the “flattening” phenomenon can be quantitatively corrected measuring the absorption spectra of the studied solution and of a solution of monomeric sCT at the same concentration [52].

After the corrections described before, CD spectra were quantitatively analyzed in agreement with the fitting procedure proposed by Whitmore and Wallace [53] and performed by the CDSSTR software available at the website of the Birkbeck College of the University of London [54]. The normalized root-mean-square deviation (NRMSD) parameter is a fit parameter, which is a measure of the difference between the experimental ellipticities and the ellipticities of the back-calculated spectra for the derived structure. It is defined as:

Σ [(θ_exp_ − θ_cal_)^2^/(θ_exp_)2]^½^, (4)

summed over all wavelengths.

### 2.6. Energy Filtered-Transmission Electron Microscopy (EF-TEM)

Negative staining is a fast method providing reliable structural information and requiring only a small amount of sample [55]. The principle involves the embedding of electron-transparent specimen within a thin amorphous layer of heavy metal salt. Negative stain was obtained by a phosphotungstate acid (PTA) 2% *w/v* solution buffered at pH = 7.3 with NaOH. To avoid salt precipitation from PTA and/or NaOH, that can be misleading interpreted as actual structures, the staining solution was filtered before each preparation through polycarbonate 0.2 μm pore filters. A droplet of the suspension containing liposomes and proteins was deposited onto 300 mesh copper grids for electron microscopy (MERCK KGaA, Burlington, MA, USA), covered with a very thin amorphous carbon film (about 20 nm) and the excess of liquid was removed by placing the grid on filter paper. When the grid was dried, a droplet of the staining solution was deposited and dried following the same procedure.

The samples were studied in a ZEISS 902 Transmission Electron Microscope (CARL ZEISS MICROSCOPY, Jena, DE), operating at 80 kV, equipped with an electron energy loss filter. In order to enhance the contrast, the microscope was used in the electron spectroscopy imaging (ESI) mode. In this configuration, the energy filter was settled to collect only elastic electrons (ΔE = 0) avoiding the contribution of inelastic electrons to the image formation. Inelastic electrons scattered from the sample contribute only to the image background noise and their filtering out strongly enhances the final image quality [56]. The heavy metal (W) surrounding the specimen to be imaged, scatters electrons more efficiently than the specimen itself, providing high image contrast and allowing a detailed examination of the structure. The attainable resolution can be evaluated in the order of 2 nm [57]. Finally, heavy metal salts give good radiation protection and maintain the structural integrity under the electron beam bombardment. The image acquisition was performed by a digital CCD camera model HSC2 (PROSCAN GMBH, Scheuring, DE; 1 k pixels × 1 k pixels) thermostatted by a Peltier cooler model WKL 230 (LAUDA GMBH, Lauda-Konigshofen, DE). Image analysis and quantification was performed by a digital image analyzer analySIS 3.0 (SIS GMBH, Munster, DE). This software allows to enhance contrast and sharpness of the acquired images and to perform morphological quantification and statistics. The dimensional measurements were performed after a careful magnification calibration of the whole imaging system based on reference standards.

### 2.7. Neurotoxicity Experiments

HT22 cells were developed from their analogous HT4 cells, immortalized from primary mouse hippocampal neurons. Cells were maintained at 37 °C, 10%, CO_2_ in Dulbecco’s modified Eagle’s medium (DMEM, Sigma Aldrich–D6546) supplemented with 10% heat-inactivated FBS and kept at less than 50% of confluence. 

Differentiation was carried out in Neuro-Basal Medium (THERMO FISHER, Waltham, MA, USA, 21103-49) containing N_2_ supplement (Gibco-17502048), at least for 24–48 h before use. Cell viability was evaluated by the 3-(4,5-dimethylthiazol-2-yl)-2,5-diphenyltetrazolium bromide (MTT) assay. Cultures were incubated for 20 min at 37 °C with 0.5 mg/mL MTT in Hank‘s balanced salt solution (LIFE TECHNOLOGIES, Carlsbad, CA, USA) and the reaction product was dissolved in dimethyl-sulphoxide. The spectral photometric absorbance of the samples was measured at a wavelength of 540 nm. The amount of MTT conversion was evaluated as percentage of the absorbance measured in treated cells compared with the absorbance of control cells.

### 2.8. Dynamic Light Scattering

The size of liposomes were measured by means of NanoZeta-Sizer (MALVERN PANALYTICAL, Malvern, UK), equipped with a 5 mW He–Ne laser. The normalized intensity autocorrelation functions were measured at an angle of 173°. The decay times were used to obtain the distribution of the diffusion coefficients D of the particles, further converted into a distribution of the effective hydrodynamic radii, RH, using the Stokes–Einstein relationship RH = kBT/6πηD, where kBT is the thermal energy and η the solvent viscosity. The values of the radii reported here correspond to the average on the “intensity weighted” distribution [58].

### 2.9. Statistical Analysis

The differences between permeabilization values (*N* = 5) of the four different groups were evaluated by ANOVA followed by a Dunnett’s test, with a confidence level of 95%. Cellular viability evaluation for each experimental condition were obtained in quadruplicate *N* = 4 and data normalized to control. A multiple comparison in cellular viability was obtained with respect to controls for all experimental conditions, by means of ANOVA followed by a Dunnett’s test, with a confidence level of 95%.

## 3. Results and Discussion

### 3.1. The sCT Solutions

To investigate the aggregation process from a morphological and structural point of view, we studied solutions representative of the three phases, which are 1 day for the “Lag-phase” (namely T1), 13 days for the “Growth-phase” (namely T13) and 20 days for the “Saturation-phase” (namely T20). We applied EF-TEM, SEC and CD techniques.

**Figure 2:**
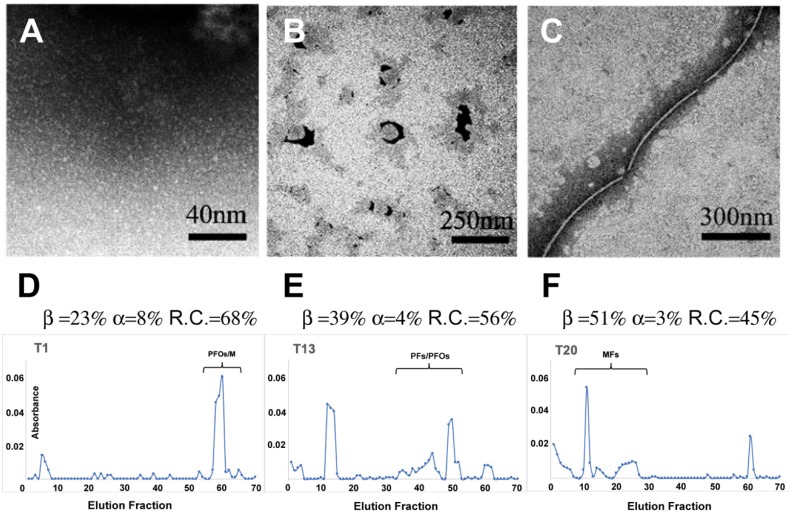
TEM images of salmon calcitonin aggregates typical of the three phases: “Lag-phase” (T1; **A**), “Growth-phase” (T13; **B**) and “Saturation-phase” (T20; **C**) together with the corresponding SEC profiles (elution fractions vs. absorbance at λ = 280 nm). The protein configuration percentages obtained by circular dichroism spectroscopy, were also reported (**D**–**F**).

EF-TEM images (Figure 2A–C) showed the protein organized in small globules (d < 10 nm) in the “Lag-phase” likely monomers and PFOs, in the “Growth-phase” rounded aggregates (d = 30 nm) likely PFs, and in the “Saturation-phase” MFs of remarkable extension together with PFs.

SEC elution profiles (Figure 2D–F) relative to these solutions showed several peaks, indicating that different populations coexisted even though one was the dominant. In particular, in the “Lag-phase” a broad peak (from monomers to hexamers, namely PFOs) had its maximum at size corresponding to dimers. In the “Growth-phase” the contribution of PFOs was strongly reduced and the spectrum dominated by peaks corresponding to aggregates of size ranging from 30 to 70 units (namely PFs). Finally, in the “Saturation-phase” all peaks previously observed were absent and strong peaks located at higher size (up to 130 units) were detected (namely MFs).

To study the evolution of the protein conformation during the aggregation process at 4 °C, we performed time-course CD experiments (t = 0, 1, 9, 15 and 21 days; Figure S1 of Supporting Materials). Along with each CD spectrum, the corresponding ultraviolet absorption spectrum was acquired to perform the “scattering” and “flattening” corrections described in section Materials and Methods. Table 1 shows results relative to the different protein configurations, obtained by the CDSSTR fitting procedure [53,54]. From the analysis of the absorption spectra used to correct the raw spectra for “flattening” and “scattering”, we found that, up to 9 day, the corrections were negligible. At 15 day, only the “flattening” correction became considerable and, at 21 day, both corrections must be applied.

We could observe that the total percentage of the β-structures (β-sheet and β-turn) systematically increased throughout the aggregation process, with the concomitant decrease of RC and α-components. Notably, in the “Lag-phase” and “Growth-phase” (up to 15 days) the majority of the sCT was in the RC configuration and only in the “Saturation-phase” (at 21 days), the majority of sCT was in β-configuration. Some years ago, we studied in detail the aggregation process of sCT at RT (13 μM in PB) obtaining an aggregation rate considerably enhanced, scaled of about 3.5 [59].

### 3.2. Functional Investigation on the Interaction of sCT with Liposomes and Neurons

To investigate the functional properties of the three representative solutions, we performed Ca^2+^-influx experiments in DPPC liposomes with and without GM1 and Chol and viability experiments on HT22-DIFF neurons.

#### 3.2.1. Ca^2+^-Influx in Liposomes

Permeabilization experiments were performed by emission fluorescence measurements, exploiting the high fluorescence intensity increase in response to Ca^2+^ binding of the dye FLUO-4, loaded in the liposome aqueous core. By monitoring the variation in FLUO-4 fluorescence emission before and after Ca^2+^ addition, we were able to evaluate the small Ca^2+^-influx induced in liposomes by the different sCT aggregates [60]. To verify the liposome integrity we performed DLS measurements before and after the sCT interaction. Results showed that all kind of liposomes maintained their shape after 24 h of treatment (Table S1 of Supporting Materials). As pointed out by Sciacca et al., high Ca^2+^ concentration can affect the protein aggregation and interaction with membranes, stabilizing small-sized oligomeric species and inhibiting pore formation [60]. To overcome this problem, we allowed sCT aggregates to interact with liposomes in the absence of Ca^2+^ and we added CaCl_2_ solution immediately before the fluorescence measurement. We decided to work at a final Ca^2+^ concentration, reached immediately after the CaCl_2_ addiction, of 172 μM suitable to maximize the method sensitivity but do not cause liposome fusion and dispersion of the dye in the bulk [61].

To investigate on the role played by the electrostatic forces present at the membrane surface, we compared results obtained with GM1-containing liposomes, DPPC/Chol/GM1 and DPPC/GM1, with those relative to the zwitterionic DPPC/Chol and plain DPPC liposomes. To investigate the effects due to the membrane fluidity, the comparison was made between Chol-containing liposomes DPPC/Chol/GM1 and DPPC/Chol, and liposomes made of DPPC/GM1 and plain DPPC. Moreover, to strengthen the hypothesis that the possible Ca^2+^-influx was due to the formation of small pores through the liposome bilayer, we performed experiments with Magainin II (Mag. II) that is a protein recognized for its ability to form small toroidal transmembrane pores [62,63].

**Figure 3:**
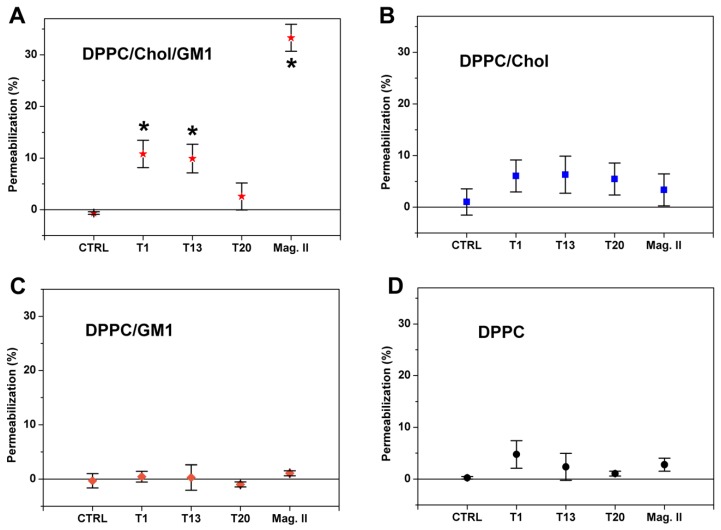
Membrane permeabilization induced by sCT solutions (13 μM) representative of the “Lag-phase” (T1), “Growth-phase” (T13) and “Saturation-phase” (T20) in the four types of liposomes: DPPC/Chol/GM1 (**A**), DPPC/Chol (**B**), DPPC/GM1 (**C**) and DPPC (**D**). The effects induced by Magainin II (Mag. II) at 13 μM in the same membrane models, are also reported. CTRL represents the permeabilization in the plain liposomes, error bars the standard errors (*N* = 5) and * *p* < 0.05.

Results showed that only in the case of the “raft” model (Figure 3A), solutions rich in partially structured PFOs (T1 and T13) and not solutions rich in structured MFs (T20) induced a small but significant permeabilization (about 10%). The absence of GM1 (Figure 3B) or Chol (Figure 3C) or both (Figure 3D) resulted in the strong reduction of permeabilization (only values relative to T1 and T13 in the “raft” system were statistically different from values relative to the other systems). Notably, the permeabilization induced by Mag. II qualitatively followed the same trend, even though the permeabilization in the “raft” model resulted to be more intense (about 30%).

Summarizing the permeabilization results, the Ca^2+^-influx induced by sCT-PFOs could be ascribed to electrostatic interactions due to the presence of the charged GM1. This was also supported by DLS results showing that the size of the GM1-containing liposomes systematically grew after 24 h of interaction with sCT (Table S1 of Supporting Materials). Moreover, the evident similarity with permeabilization induced by Mag. II supports the idea that the electrostatic interaction drove the pore formation. In fact, Mag. II formed transmembrane pores in the presence of negatively charged lipids (phosphatidylglycerol) and at a rate decreasing with the increase of the membrane packing [62,63]. In our case, Chol enhanced membrane fluidity and its absence strongly reduced permeabilization. The role played by Chol in the amyloid pore formation has been proposed in literature [41,42] and, recently, Meleleo and Sblano showed that the Chol promoted human CT incorporation and channel formation in planar lipid membranes [64].

#### 3.2.2. Neurotoxicity

We studied the neurotoxicity of the three representative solutions testing their biological activity on HT22-DIFF neuronal cells. To investigate the nature of the damage, we made a comparison with Mag. II, a protein well-known to permeabilize membranes by forming pores [62,63] and we used the vehicle solution (PB at 5 mM) as a negative control.

**Figure 4:**
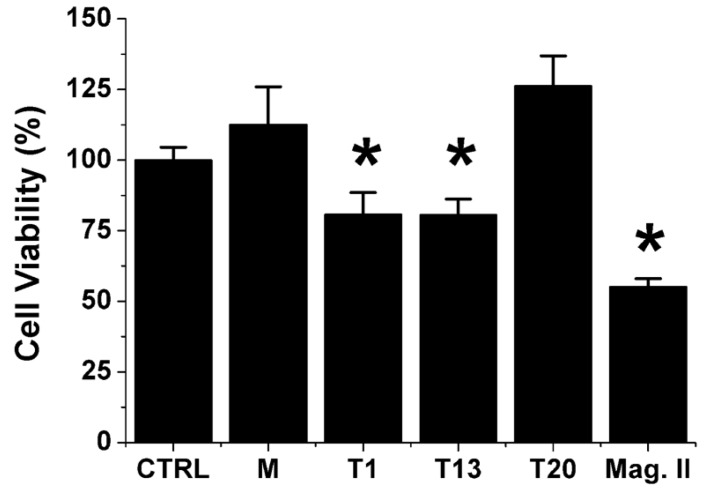
Neurotoxicity results obtained in HT22-DIFF neuronal cells, relative to sCT solutions representative of the “Lag-phase” (T1), “Growth-phase” (T13) and “Saturation-phase” (T20). Result relative to Magainin II (Mag. II) is also reported. CTRL represents the treatment with the vehicle solution (PB at 5 mM). Error bars represent the standard error (*N* = 4) and * indicate *p* < 0.05.

Results (Figure 4) clearly indicated that solutions representing the “Lag-phase” and the “Growth-phase”, both characterized by the RC configuration, were neurotoxic while the one relative to the “Saturation-phase”, with sCT in β-configuration, were totally inactive. Mag. II induced the strongest biological effects. 

Notably, in our previous paper concerning neurotoxicity in hippocampal neurons treated by sCT-PFOs we showed that, under physiological conditions, other cellular mechanisms were involved [20]. Nevertheless, in the same study we showed that the Ca^2+^-influx due to membrane permeabilization was the triggering factor that induced NMDA-mediated neurotoxicity and this is in good agreement with the results obtained now with both HT22-DIFF cells and liposomes.

Summarizing all functional results, by incubating sCT solutions with liposomes mimicking “lipid-rafts” and neurons for 24 h, in the “Lag-phase” we obtained considerable Ca^2+^-influx and neurotoxicity. In the “Growth-phase”, we observed the same behavior while, in the “Saturation-phase”, we obtained totally inactive solutions. Permeabilization clearly indicated that the absence of GM1 or Chol or both strongly reduced or abolished the Ca^2+^-influx.

### 3.3. Structural Investigation on the Interaction of sCT with Liposomes Mimicking “Lipid-Rafts”

To unravel the structural changes occurring during the interaction, we performed combined experiments of CD and EF-TEM on liposomes mimicking “lipid-rafts”, the only ones affected by PFOs.

#### 3.3.1. Protein Conformation Evolution by CD Spectroscopy

**Figure 5:**
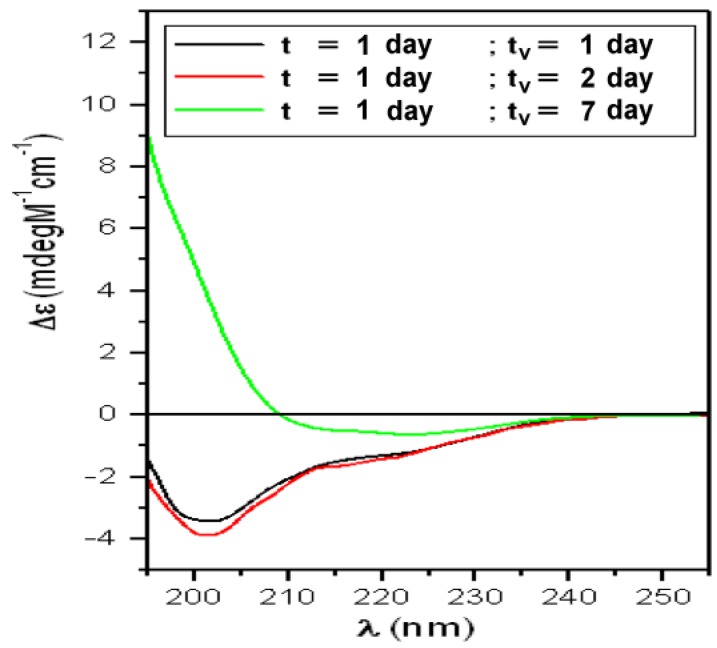
CD spectra relative to sCT solution representing the “Lag-phase” (T1) in the presence of liposomes incorporating GM1 and Chol mimicking “lipid-rafts”. The interaction was studied at room temperature and increasing lifespan (t_v_).

Spectra obtained after the interaction, at RT, of the solution representing the “Lag-phase” (T1) with liposomes mimicking “lipid-raft” are reported in Figure 5 and the fitting results in Table 2. The analysis revealed that the “scattering” correction was ineffective while the “flattening” slightly modified all spectra.

Before the interaction, this solution was characterized by a population rich in partially structured PFOs (α = 8%; total β = 23%%; RC = 68%; Table 1). After 1 day of interaction, the β-components quickly increased up to 35% and after one week, were doubled (67%). This demonstrates that GM1 foster the binding of partially structured PFOs to the lipid bilayer and induces a fast β-conformation rise, in agreement with results reported in our previous paper where we studied this phenomenon by Langmuir planar membrane models [47]. Moreover, we note that the β-configuration (35%) after 24 h of interaction, was equal to that we obtained (38%) in a previous paper were we reconstituted sCT in the bilayer during the liposome preparation [65].

**Figure 6:**
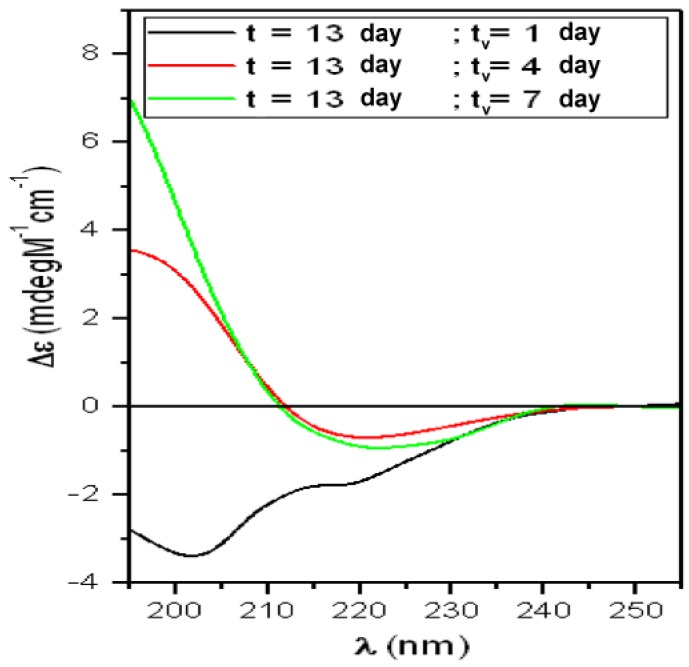
CD spectra relative to the sCT solution representing the “Growth-phase” (t = 13 days) in the presence of liposomes incorporating GM1 and Chol mimicking “lipid-rafts”. The solution was studied at room temperature and increasing lifespan (t_v_).

CD spectra obtained after the interaction, at RT, of the solution representing the “Growth-phase” (T13) with liposomes mimicking “lipid-raft” and corrected for the “flattening” are reported in Figure 6 (fitting results in Table 3). For the first time, the “scattering” correction gave considerable effect on the spectrum relative to 7 days, suggesting that particles of considerable dimension were present. As stressed in the Section 2.3, in this case the fitting procedure gave only qualitative percentages.

Before the interaction, this solution was characterized by a mixed population of PFOs and PFs but the majority of proteins was in the RC configuration (α = 4%; total β = 39%%; RC = 56%; Table 1). This indicated that the partially structured PFOs were still dominant even though a higher percentage of β-structures (39%) suggested that PFs were growing. After 1 day of interaction, we obtained conformation percentages (Table 3) similar to those obtained for the “Lag-phase” (Table 2) and the same for permeabilization results (Figure 3). We interpreted these outcomes as due to the inefficacy of PFs and action of the partially structured PFOs, still dominant in the solution.

After 4 days of interaction the β-structure percentage became very high (70%) and this trend was maintained up to 7 days. This can be interpreted as due to the binding of the preformed PFs to the lipid bilayer driven by GM1, and their subsequent aggregation. This is in agreement with results published by Matsuzaki about the role played by membranes in the amyloidogenesis for Aβ [37]. In this paper, GM1 clusters promoted the binding of monomers and the formation of aggregates of 15 molecules in β-sheet. In the pathologic state, the Aβ concentration was high and those aggregates were seed for fiber growing.

**Figure 7:**
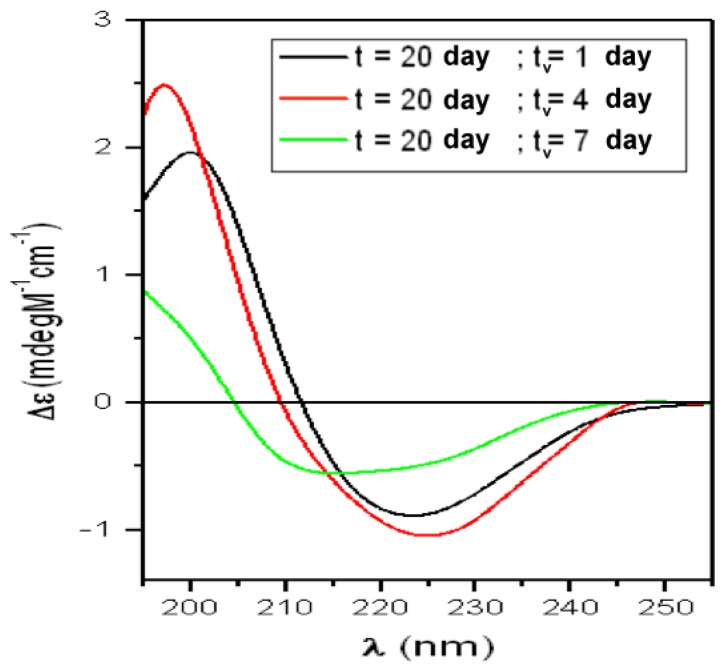
CD spectra relative to the sCT solution representing the “Saturation-phase” (t = 20 days) in the presence of liposomes incorporating GM1 and Chol mimicking “lipid-rafts”. The solution was studied at room temperature and increasing lifespan (tv)

CD spectra obtained after the interaction, at RT, of the solution representing the “Saturation-phase” (T20) with liposomes mimicking “lipid-raft” and corrected for the “flattening” are reported in Figure 7 and results of the fitting in Table 4. As discussed in Section Materials and Methods, due to the impossibility to perform a quantitative “scattering” correction, they must be considered only qualitative indications.

This solution was characterized by a population rich in MFs, with the majority of sCT in β-structures (α = 3%; total β = 51%; RC = 45%; Table 1). It is interesting to note that, just after 1 day of interaction, the β-structures were at the maximum measured value (72%) and this trend was maintained up to 7 day. This suggests that well-formed MFs were adsorbed to the lipid bilayer and rapidly aggregated.

#### 3.3.2. Liposome Morphological Evolution by EF-TEM

To follow, at the molecular resolution, the structural changes occurring during the early stages of interaction, we applied EF-TEM to liposomes mimicking “lipid-raft”, the only ones affected by PFOs. We studied solutions representative of the three phases, after 1 day of interaction.

**Figure 8:**
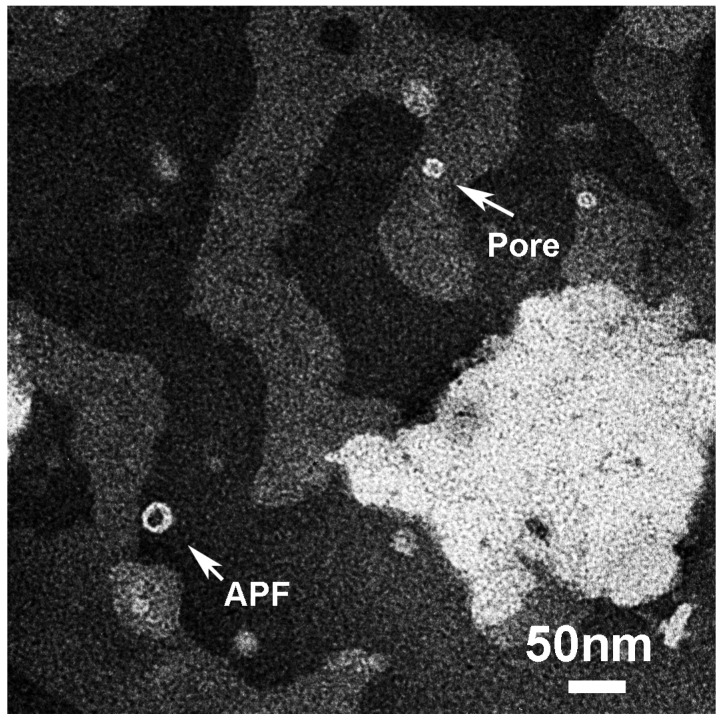
Energy-filtered (EF)-TEM images showing liposomes (gray) incorporating GM1 and Chol mimicking “lipid-rafts”, collapsed onto the amorphous carbon substrate (black). Small and geometrically ordered pore-like structures (white) can be observed in liposomes together with annular protofibrils (APFs) located outside.

Low magnification micrographs of liposomes interacting with the solution representing the “Lag-phase” (T1; Figure 8) showed collapsed liposomes that can be clearly recognized as gray islands in the black substrate, together with white donuts located in liposomes or onto the black substrate.

**Figure 9:**
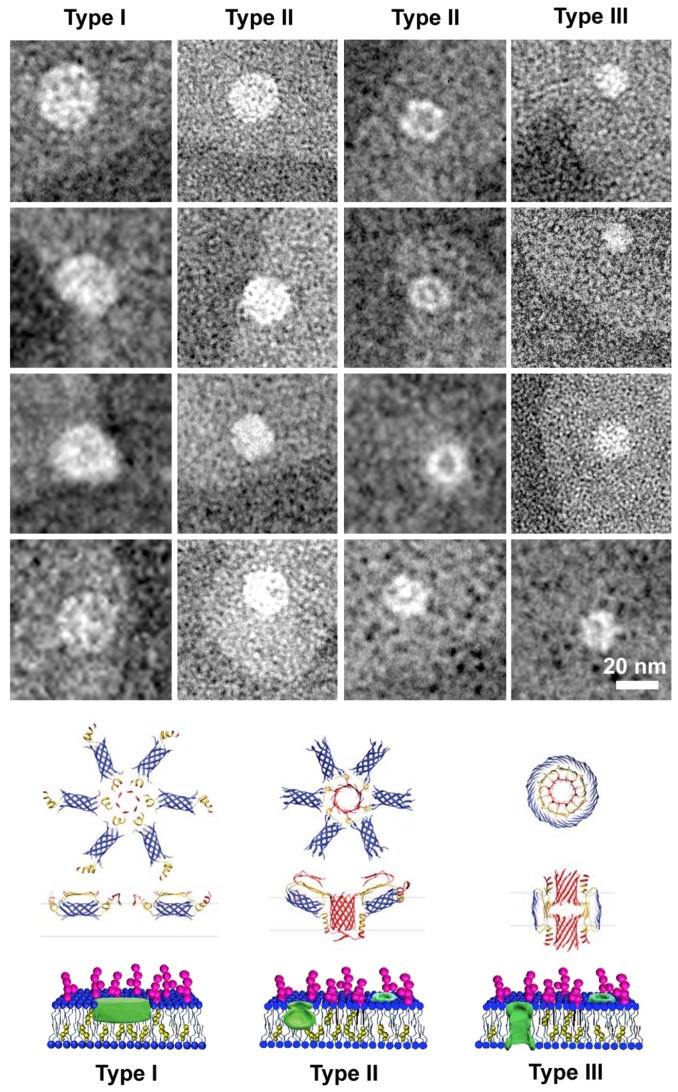
Membrane-bound sCT assemblies visualized by EF-TEM (top) organized in tree decreasing size types, compared with the model proposed by Shafrir et al. for Aβ [44] (middle). Type I represents the formation of hexameric patches at the surface, Type II their tilting due to the incorporation in the membrane (in the right column the central pore can be observed) and Type III the well-formed membrane pore. A possible interpretation based on the neutron reflectometry study of Rondelli et al. [66], is also reported (bottom).

Aggregates located in the liposomes, imaged at high resolution in Figure 9, were characterized by a hexagonal symmetry and of three different sizes (Type I, II and III). They could represent the direct visualization of the “amyloid-pores” proposed by Lashuel and Lansbury [21]. GM1-free liposomes appeared less collapsed and didn’t show any similar structure at the surface (Figure S2 of the Supporting Materials).

Notably, Shafrir et al. proposed a MD simulation model to identify structural motifs for Aβ assemblies, stable in the membrane environment. Hexameric aggregates preformed in solution, namely “beads” (PFOs in our paper) were hypothesized to bind the membrane and subsequently to further assemble in complex structures able to span the bilayer. Once such aggregates of several hexamers have spanned the bilayer, they may merge to form a stable β-structured pore [44].

They proposed several stages of pore formation (middle part of Figure 9) that well match our EF-TEM observations for sCT. In the first stage, hexamers interacted with the membrane forming surface patches of hexagonal symmetry, very similar to our sCT aggregates (Type I in Figure 9). The second interaction stage, when tilted hexamers started membrane internalization, could be represented by Type II assemblies where the hexagonal symmetry was preserved and size reduced (the central pore formation can be also observed). Finally, the pore spanning the bilayer can be represented by Type III smaller aggregates. Structures were compatible with a hexagonal symmetry, in good agreement with the Shafrir’s model, but the central hole was not easily observed likely due to lack of resolution or incomplete staining.

Features imaged in our EF-TEM study were bigger that that proposed by Shafrir et al. [44]. However, it is known that several factors (peptide kind, concentration and lipid composition of the membrane) can influence structure and dimension of amyloid assemblies in the membrane. Notably, the Shafrir’s simulation has been performed without GM1 and Chol that are present in our experiments. Meleleo and Sblano, working with human CT that forms pores in planar membranes, concluded that their dimensions increased in the presence of Chol [64].

**Figure 10:**
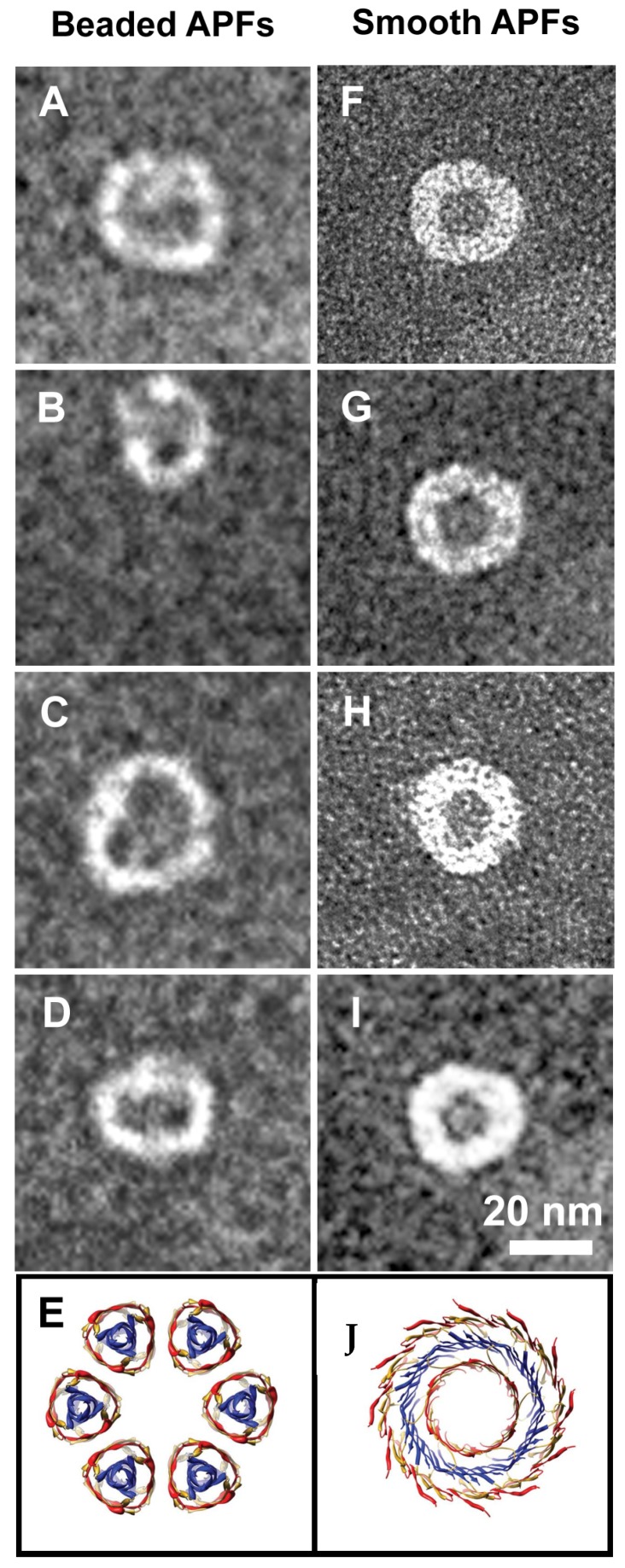
The two kinds of sCT-APFs observed outside liposomes: “beaded” (**A**–**D**) and “smooth” (**F**–**I**). For comparison, the molecular structures obtained by molecular dynamics simulation by Shafrir et al. for Aβ [43], are also shown (**E**,**J**).

The applicability of the Shafrir’s model is further supported by APFs we found outside liposomes in the same solution, imaged at high magnification in Figure 10. We visualized two types of APFs: “beaded” and “smooth”. “Beaded” have been described in the literature for Aβ as formed by a ring of spherical particles of 3–5 nm in diameter while “smooth” were continuous and thicker [12].

For comparison with our sCT-APFs, we reported in Figure 10E,L the structures simulated by the Shafrir’s model [43]. In the Shafrir’s model each “bead” was formed by an hexameric assembly (PFOs) that assemble to form a “beaded” APF (Figure 10E) to eventually merge to form a continuous “smooth” APF (Figure 10L) [44]. We note that the size of the beads forming the “beaded” sCT-APF (about 6 nm) was comparable with hexamers even though actual assemblies were more complicated. Conversely, “smooth” APFs seemed more homogeneous and similar to the Shafrir’s model.

However, Shafrir et al. simulated the interaction of the hexameric assemblies with model membranes in the absence of GM1 and Chol and our experimental data and other indicated that these two molecules played a pivotal role.

The hypothesis of a GM1-driven interaction between PFOs and membranes is supported by the findings of Rondelli et al., who performed a neutron reflectometry study on raft-mime GM1- and Chol-containing model membranes interacting with structured or partially structured Aβ early-oligomers [66]. Notably, they concluded that partially structured oligomers were captured by the membrane and deeply dig it towards the opposite side. Their organization in the lipid bilayer was templated by the membrane into a forming pore (Figure 9).

Very long all-atoms MD simulations were used to investigate the behavior of Aβ monomers and dimers, characterized by the α-helical conformation, in GM1-containing raft-like membrane. Results showed that the carbohydrate headgroup of GM1 acted as binding site for Aβ and induced a β-hairpin structure at the C-terminus of the peptide [67].

We note that our CD data showed that, in our sCT-PFOs, we had 28% of β-structure (Table 1). After the interaction with the membrane mimicking the “lipid-rafts” our data indicated that the β-component increased (from 23% to 35%) while the α-helical component did not change (from 6% to 8%; Table 2). This suggests that, in our case, the formation of sCT-PFOs occurred outside the bilayer and before the interaction, in line with the model of Shafrir et al. [43].

Computational studies concerning the effect of Chol in the protein–lipid interaction have been also performed. In particular, the stability of an annular channel in a membrane-like matrix has been studied by Di Scala et al. [41]. In this study, the oligomeric channel consisted of eight Aβ/Chol subunits in which the peptide was in a tilted α-helical conformation. In another study, Pannuzzo investigated the propensity of Aβ monomers to self-assemble and to form circular assemblies into a lipid bilayer [42]. He found that structure and stability of the aggregates into the membrane was sensitively correlated to the presence and arrangement of Chol. Both studies indicated that the protein aggregation and pore formation occurred inside the membrane, leading to α-helical conformation. This scenario is in contrast with our CD results that did not show any α-helical increase after the interaction (from 8% to 6%; Table 2).

Our structural data support the Shafrir’s model where the pore formation occurs inside the bilayer after the incorporation of hexamers (PFOs) formed outside and leads to an increase of the β-structures. Chol allows this mechanism making the rigid membrane, made of saturated DPPC and GM1, more fluid [29] and allowing hexamers to be incorporated.

**Figure 11:**
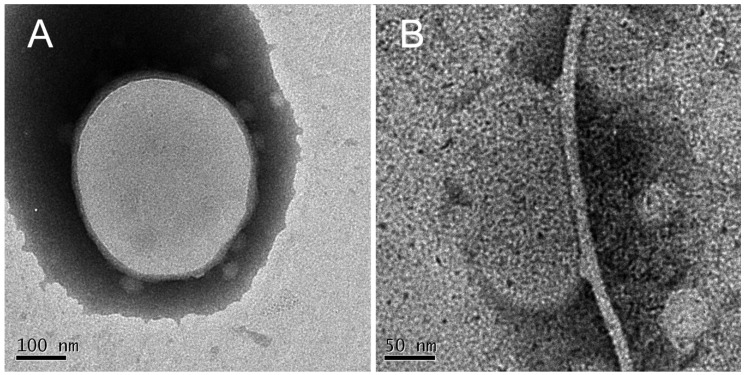
EF-TEM micrograph representing a liposome incorporating GM1 and Chol mimicking “lipid-rafts” interacting with PFs (solution T13; **A**) and with a long and thin MFs (solution T20; **B**) without membrane disruption or internalization.

EF-TEM images relative to liposomes interacting with the solution representing the “Growth-phase” (T13; Figure 11A) showed the rounded aggregates (PFs) of about 30 nm, just observed in the sample representative of the “Growth-phase” (Figure 2B), adsorbed to the external liposome face, without membrane disruption. Images relative to liposomes interacting with the solution representing the “Saturation-phase” (T20; Figure 11B) revealed the presence of big and long MFs in contact with liposomes.

Summarizing all our structural results, we note that the presence of GM1 acted as a binding site for partially structured PFOs increasing their β-structure component after the interaction with the membrane, as indicated by CD results. We speculated that, in a first step the negatively charged moiety of GM1 bound the positive parts of sCT molecules of PFOs. This can happen only when a suitable PFO structural arrangement exists: the sCT positive parts are exposed to the solvent. In our interpretation of CD data (Table 2), in the PFO outside the bilayer the 23% of molecules forms a hydrophobic β-core and the remaining part (68%) a positive shell. This is due to the tendency to expose the polar residues towards the solvent with the aim to maximize the distance between its polar parts. In a second step, the minimization of the hydrophobic mismatch between the PFO hydrophobic core and the bilayer backbone leads to its incorporation, with the final formation of a transmembrane “amyloid-pores” as proposed by Shafrir et al. [44]. As imaged by EF-TEM, PFs and MFs are also attracted by GM1, but grow on the liposome external face without membrane internalization.

## 4. Conclusions

**Figure 12:**
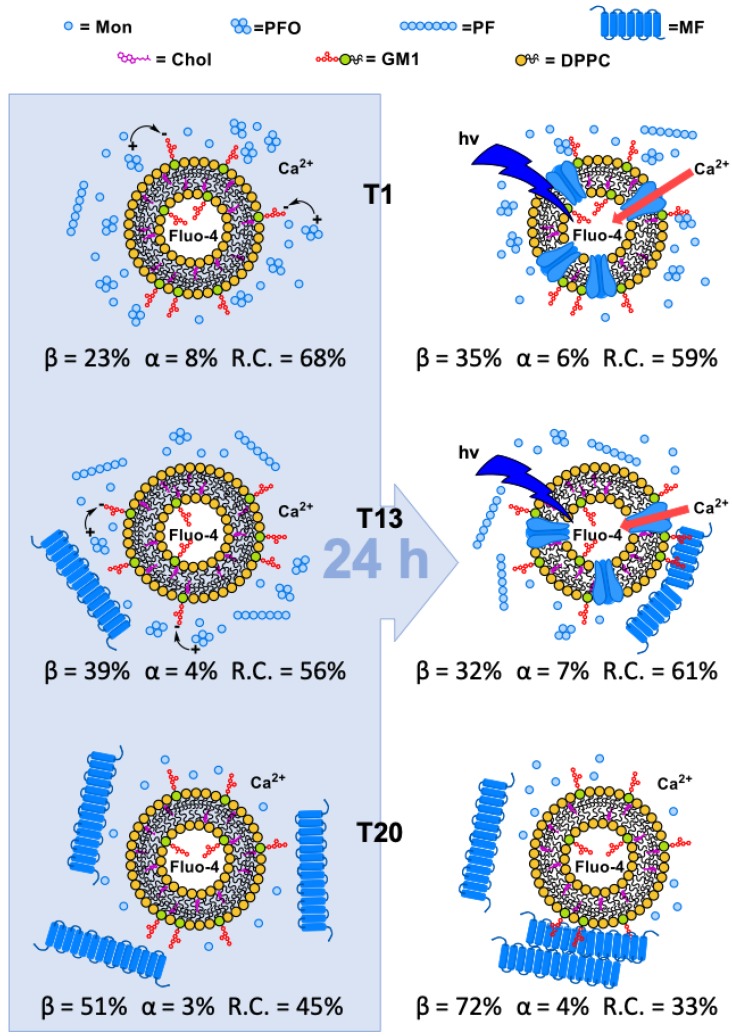
Graphical sketch summarizing the interpretation of the all body of our experimental data.

The all body of our results, concerning experiments performed by several biophysical techniques on liposomes mimicking “lipid-rafts” in the presence of sCT aggregates, demonstrated that the negatively charged GM1, embedded in the bilayer, triggered the interaction of the aggregates with this protein (Figure 12). In particular, partially structured and mainly disordered PFOs resulted to be able to damage the lipid bilayer by the formation of “amyloid-pores” and this was made possible by Chol that rendered fluid membranes. This is in good agreement with the biological results we recently published about Ca^2+^-influx induced damage in hippocampal primary neurons [20] and allows us to speculate about the molecular mechanisms at the basis of neurotoxicity.

Permeabilization results clearly indicated that only solutions where metastable PFOs and not stable MFs were dominant damaged membranes mimicking “lipid-rafts” inducing Ca^2+^-influx. The lack of GM1 and Chol or both dramatically reduced the Ca^2+^-influx. In good agreement, neurotoxicity results showed that only solutions rich in PFOs and not MFs affected cell viability.

Structural results gave interesting information about the molecular mechanisms. On the one hand, CD indicated that GM1 triggered the interaction between the “lipid-rafts” and the preformed PFOs, speeding up the process of β-structure formation. On the other hand, EF-TEM showed that only PFOs were able to damage “lipid-rafts”, forming “amyloid-channels” very similar to that previously described for Aβ. Conversely, PFs and MFs grew outside the bilayer.

For what concerns the nature of the forces driving the interaction, we speculated that the electrostatic force was responsible for the first contact of PFOs with the membrane while the hydrophobic force for the subsequent incorporation performed to minimize the hydrophobic mismatch [18,21,22]. This scenario is in good agreement with the model proposed by Shafrir et al. with MD simulation, leading to the formation of “amyloid-pores” starting from preformed partially ordered hexamers [44]. Only partially ordered PFOs that have the suitable flexibility and hydrophobic profile were able to penetrate the bilayer, as proposed by Campioni et al. [14].

In conclusion, two structural factors of the PFO seem to be decisive to render it active: (i) the surface charge, positive at physiological pH; (ii) the partially ordered structure, flexible and with an external amphipathic profile. Regarding membranes, “lipid-rafts” seem to be the natural PFO target and the exposition of negatively charged moieties combined with the presence of Chol, the necessary condition to form “amyloid-pores”. Notably, these factors seem to be common to several studies concerning different amyloid proteins and this fact supports the intriguing hypothesis of the existence of a common mechanism for the amyloid pathogenesis.

## Figures and Tables

**Table 1:** Percentage of the secondary structures obtained by the fitting procedure, relative to CD spectra relative to sCT incubated at 4 °C for increasing times (t; see Figure S1). The normalized root-mean-square deviation (NRMSD) value (last column) represents an estimation of the goodness of fitting procedure (see Section 2).

t (Day)	α-Helix (%)	β-Sheet (%)	Turns (%)	Random-Coil (%)	NRMSD
0	6	15	11	69	0.021
1	8	13	10	68	0.025
9	4	19	14	61	0.026
15	4	24	15	56	0.031
21	3	32	19	45	0.054

**Table 2:** Percentage of the sCT secondary structures obtained by the CD spectrum relative to sCT representing the “Lag-phase” (T1) solution, before and after the interaction with DPPC/Chol/GM1 (spectra of Figure 5).

T1	α-Helix = 8%	Total β-Structures = 23%		Random-Coil = 68%	
		t = 1 day “Lag-phase”			
t_v_ (day)	α-Helix (%)	β-sheet (%)	Turns (%)	Random-Coil (%)	NRMSD
1	6	20	15	59	0.024
2	6	20	13	60	0.018
7	3	53	14	30	0.005

**Table 3:** Percentage of secondary structures obtained by the fitting procedure, relative to spectra of Figure 6.

T13	α-Helix = 4%	Total β-Structures = 39%		Random-Coil = 56%	
t = 13 days“Growing-Phase”					
t_v_ (day)	α-Helix (%)	β-Sheet (%)	Turns (%)	Random-Coil (%)	NRMSD
1	7	18	14	61	0.018
4	1	48	22	28	0.014
7	6	47	23	24	0.004

**Table 4:** Percentage of secondary structures obtained by the fitting procedure, relative to spectra of Figure 7.

T20	α-Helix = 3%	Total β-Structures = 51%		Random-Coil = 45%	
t = 20 days “Saturation-Phase”					
t_v_ (day)	α-Helix (%)	β-sheet (%)	Turns (%)	Random-Coil (%)	NRMSD
1	4	48	24	33	0.019
4	4	33	19	43	0.004
7	7	32	22	40	0.064

## Supplementary Materials

The following are available online at https://www.mdpi.com/2218-273X/10/1/58/s1. Figure S1: sCT configuration during aggregation, Figure S2: GM1-free liposome morphology, Table S1: Liposome sizes before and after treatments.

## Abbreviation List

Salmon Calcitonin (sCT); Prefibrillar-Oligomers (PFOs); Proto-Fibers (PFs); Mature-Fibers (MFs); Monosialoganglioside-GM1 (GM1); 1,2-Dipalmitoyl-*sn*-glycero-3-phosphocholine (DPPC); Cholesterol (Chol); Circular Dichroism (CD); Energy Filtered-Transmission Electron Microscopy (EF-TEM); Amyloid-β (Aβ); α-Synuclein (αS); Amylin (hIAPP); Prion (Pr); Random Coil (RC); Annular-Protofibrils (APFs); Linear-Protofibrils (LPFs); Alzheimer’s Disease (AD); Molecular Dynamics (MD); Size Exclusion Chromatography (SEC); Room Temperature (RT); Hexafluoroisopropanol (HFIP); Phosphate Buffer (PB); Magainin II (Mag. II); Dynamic Light Scattering (DLS); Normalized Root-Mean-Square Deviation (NRMSD); Phosphotungstate Acid (PTA); 3-(4,5-dimethylthiazol-2-yl)-2,5-Diphenyltetrazolium Bromide (MTT).
