# Supplementary material for: The Interaction between Amyloid Prefibrillar Oligomers of Salmon Calcitonin and a Lipid-Raft Model: Molecular Mechanisms Leading to Membrane Damage, Ca2+-Influx and Neurotoxicity
Source: Biomolecules. 2019 Dec 29;10(1):58. doi: 10.3390/biom10010058 (PMC7022306; doi:10.3390/biom10010058)
Supplement: Supplementary file 1 [file biomolecules-10-00058-s001.pdf]

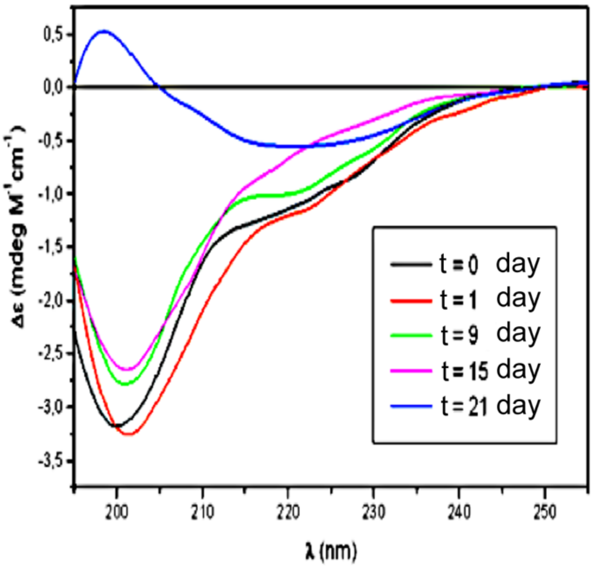

Figure S1: sCT configuration during aggregation. CD spectra relative to plain sCT solution (13 μM) incubated in PB at 4 °C for increasing lifespan (**t**).

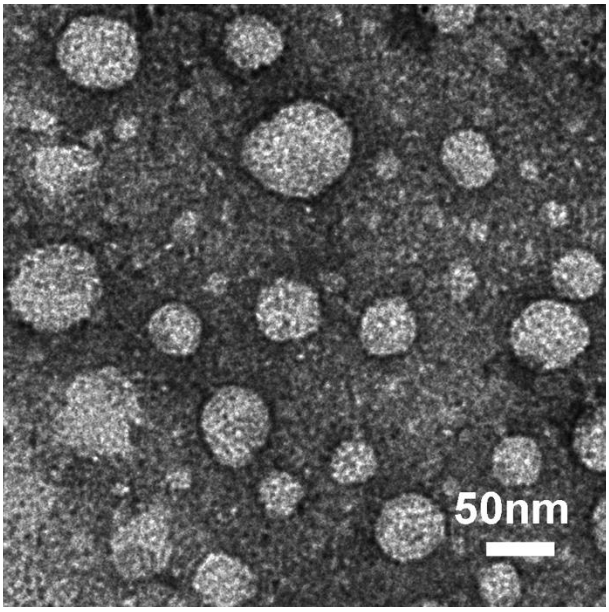

Figure S2: GM1-free liposome morphology. Typical EF-TEM image of GM1-free liposomes without evident pores.

Table S1: Liposome sizes before and after treatments. DLS results relative to the hydrodynamic diameter of liposomes before and after the sCT treatments.

Sample	DPPC/Chol/GM1	DPPC/Chol	DPPC/GM1	DPPC
CTRL	125 ± 18	123 ± 19	140 ± 25	126 ± 24
T1	178 ± 49	125 ± 22	149 ± 31	128 ± 24
T13	157 ± 31	123 ± 19	151 ± 31	130 ± 25
T20	163 ± 40	123 ± 20	157 ± 35	128 ± 26
